# Calcium Phosphate Cement Causes Nucleus Pulposus Cell Degeneration through the ERK Signaling Pathway

**DOI:** 10.1515/biol-2020-0021

**Published:** 2020-05-06

**Authors:** Quan Zhou, Cenhao Wu, Jiali Zha, Jun Ge, Qi Yan, Yingjie Wang, Dawei Song, Jun Zou

**Affiliations:** 1 Department of Orthopaedic Surgery, The First Affiliated Hospital of Soochow University, 188 Shizi St, Suzhou, Jiangsu 215006, China; 2 Department of Orthopaedic Surgery, The Affiliated Huai'an Hospital of Xuzhou Medical University, Huai’an, Jiangsu 223002, China

**Keywords:** calcium phosphate cement (CPC), bone cement leakage, ERK signaling pathway, cell growth and cell cycle, human nucleus pulposus cells’ degeneration

## Abstract

While calcium phosphate cement (CPC) is recognized as one of the most likely substitutes for the conventional Polymethylmethacrylate (PMMA), there are very few studies about its intradiscal leakage consequences. Herein, the goal of our study was to examine the effect of CPC particles on the ERK (extracellular regulatory kinase) pathway in human nucleus pulposus cell (HNPC) degeneration. Different concentrations of CPC particles (0.00‰, 0.01‰, 0.05‰, 0.1‰ v/v) were added to human nucleus pulposus cell cultures. After 10 days of treatment, HNPC biological behaviors and degeneration degree were analyzed by CCK-8 assay, crystal violet staining, flow cytometer and western blot. The effect of CPC on the ERK pathway was also analyzed by western blot. After activating the ERK path by overexpressing Ras, HNPCs’ biological behaviors and degeneration degree were analyzed again. We found that CPC particles had a negative effect on human nucleus pulposus cells (HNPCs), which are mainly reflected in cell growth and the cell cycle. After activation of the ERK signaling pathway, the negative effects of CPC on cell growth and the cell cycle were significantly reduced and the degeneration degree of HNPCs was reversed. CPC particles can probably block the activation of the ERK pathway, thus causing the HNPCs’ degeneration.

## 1 Introduction

Given the current aging population trend, the incidence of osteoporotic vertebral compression fracture (OVCF) can be gradually increased. Studies have shown that this kind of fracture has occurred in 20% of people over 70 and in 16% of postmenopausal women [1]. Patients who experience osteoporosis have poor vertebral strength, which can cause compression fractures after a slight injury or even no obvious trauma. Statistics indicate that the mortality rate in 5 years could reach 23%-34% when multiple segments of vertebra are involved because of the fracture itself and its serious complications of other systems, including respiratory and digestive systems [2]. Percutaneous vertebroplasty (PVP) and kyphoplasty (PKP) are two novel and minimally invasive techniques that can relieve patients’ pain, restore the vertebral height to some degree and increase patients’ mobility in the early phase by injecting the bone cements into the compressed vertebral body [3].

Polymethylmethacrylate (PMMA) and calcium phosphate cement (CPC) are two of the most common cements used for PVP and PKP. CPC is recognized as one of the most promising substitutes for the conventional PMMA because of its bioabsorbability, lower heat release during solidification and its osteoconductivity [4, 5]. What’s more, studies have shown that the long-term outcomes between CPC and PMMA after PKP of OVCF have no significant differences [6]. However even such a promising substitute should include consideration of the complications of PVP/ PKP, in which bone cement leakage is the most common [7, 8]. However, intradiscal cement leakage is ‘rare’ relative to other kinds of leakage, such as the epidural leakage and the paravertebral leakage [9]. There has already been much discussion about the serious consequences of the cement leakage, for example, the neurological symptoms when the nerve root or the spinal cord is involved [10, 11]. Clinical studies have shown that the intradiscal leakage occupies about 30.5% and 38% of the total leakage rate of PVP and PKP, respectively [12]. The total asymptomatic leakage rate of PVP and PKP is about 41-75% and 9-14%, respectively [12, 13]. Therefore, the intradiscal cement leakage is not so ‘rare’ as we used to think, but it is indeed of less concern than other kinds of leakage because it is usually asymptomatic in the short term. However, this doesn’t mean it does no harm. Though the likelihood of intradiscal cement leakage increasing the incidence of the adjacent vertebral fracture is not known [14], there have already been studies about PMMA that broke the normal structure of intervertebral disc due to its thermodynamic properties [15]. While scientists have done some research in cytological experiments of PMMA particles, the specific mechanism about whether or how exactly bone cement particles affect nucleus pulposus cells (NPCs) still remains unknown [16]. The ERK signaling pathway, also known as Ras/Raf/MEK/ERK signaling cascades, is one of the classic MAPK (mitogen activated protein kinase) signaling pathways. It can be widely activated compared with other MAPK signaling pathways. What’s more, the activated ERK participates in the process by which extracellular signals are transmitted to the nucleus through phosphorylation, and regulates the IEG (immediate early genes) expression that can promote cell proliferation and differentiation [17, 18]. There have already been some studies focusing on the role of the ERK signaling pathway in NPCs’ biological behaviors [19, 20, 21]. The goal of our study is to examine the effect of CPC particles on the ERK pathway in human nucleus pulposus cells. We speculate that CPC causes the HNPCs’ degeneration by inhibiting the ERK signaling pathway. We carried out this study to explore whether and how CPC particles affect HNPCs biologically.

## 2 Methods

### 2.1 Cell resuscitation and culture

The human nucleus pulposus cell line was obtained from ScienceCell (Carlsbad, CA, USA, Cat. No. 4800). The freezing tube was taken out from liquid nitrogen and put in the water bath under 37°C with gently shaken for 1-2 minutes until it dissolved completely. The frozen storage fluid was then gently blown and moved to the centrifuge tube. After centrifugation with 5 ml culture medium (HyClone DMEM/F12, SH30023.01B), the supernatant was removed. Subsequently, the cell suspension was moved to a culture flask in the incubator at 37°C with 5% CO_2_. The cell culture was changed after 24 hours and the changing interval was 2 days from then on. When the cell adherence rate was up to 90%, cells were passaged at a ratio of 1:2.

### 2.2 Build Ras over expression nucleus pulposus cells

The pBabe-Ras plasmid was modified. HA tag was added on the N side of the Ras open reading frame. The carrier was puromycine-resistant and the transfected cells could be selected and enriched by applying puromycin in culture medium. After the plasmid was transfected by calcium phosphate and packaged by virus, the virus then affected HNPCs. HNPCs that can express stably were selected.

### 2.3 Group of experiments

The second generation of a human nucleus pulposus cell line was selected and different concentrations of CPC (0.01‰, 0.05‰, 0.1‰ v/v) were added into the cell cultures. These cell cultures served as experimental groups, and those containing no added CPC served as the control group.

### 2.4 Cell viability analysis

Cell viability was analyzed by the WST tetrazolium salt method (CCK-8, Dojindo). The measurement procedure was as follows: 10 μL of Cell Counting Kit-8 (CCK-8) solution (Dojindo, Tokyo, Japan) was added to each well of the 96-well plates (washed twice by preheated PBS) followed by incubation at 37°C and 5% CO_2_ for 1 h. After incubation, the supernatants were carefully aspirated. To lyse the cells and completely dissolve the precipitates, 100 μL dimethyl sulfoxide (DMSO) was added to each well and the plates were shaken for 15 min. The absorbance at 450 nm was measured and the background absorbance of the medium in the absence of cells was subtracted. The experiments were independently repeated at least three times.

### 2.5 Cell cycle distribution analysis

Cell medium was collected and digested by trypsin enzyme. Then the cell medium was centrifuged at the speed of 3000rpm for 5 min. After the cells were collected, they were washed by pre-cooling PBS. They were then centrifuged again and the supernatant was removed. Subsequently, cells were fixed by 70% alcohol solution at 4°C for one night. The cells were then collected through an additional centrifuge. After the solution containing RNase (50 ng/ml) and propodium iodide (PI) staining solution (20 ng/ml) was prepared, cells were digested and put into the water bath under 37°C in the dark for 1 hour. Finally, cells were analyzed by flow cytometer.

### 2.6 Western blot

Cells were collected from each group by washing three times with PBS. The total protein was extracted by adding ice-cold RIPA lysis buffer in the Bio-Rad protein assay kit (Bio-Rad, Hercules, CA, USA). The protein was transferred to the nitrocellulose membrane after 12% SDS-PAGE electrophoresis. Then the membranes were blocked with tris-buffered saline (TBS) and Tween-20 (Thermo Fisher Scientific, Waltham, MA, USA) containing 5% fat-free-milk for 1 hour at room temperature. After the primary antibody was added, it was incubated overnight at 4°C. Primary antibodies included: anti-Bcl-2, anti-Bax, anti-Cleaved Caspase 9 (Santa Cruz, Dallas, TX, USA), anti-ERK1, anti-Phospho-ERK anti-Collagen II, anti-β-actin, anti-GAPDH (Abcam, Cambridge, MA, USA), and anti-HA-Tag (Thermo Fisher Scientific, Waltham, MA, USA). After the membranes were washed the next day, the secondary antibody (Bioworld) was incubated for 1 hour at room temperature and PBST washed again. ECL chemiluminescent substrates were added to it and X-ray was used to expose. The relative expression of the target protein was evaluated by comparing the gray value ratio of target protein content to the β-actin content (target protein/β-actin) using the Quantity One 4.62 image analysis software.

### 2.7 Cell growth analysis

Cell growth can be assessed by crystal violet staining. After digestion of cells (The state of cells should be carefully observed before digestion to ensure that the degree of cell fusion is about 50%. Too high or too low cell density is not suitable for crystal violet test.), the cells were inoculated in a 12-well plate, and each cell type was inoculated with 3 Wells, and 2000 cells were inoculated in each hole. The final volume of medium for each hole was 2 ml. The fluid was changed every other day and incubated for 7-10 days. Subsequent steps involved discarding the medium, adding 2 ml crystal violet solution, and staining for 10 minutes. Crystal violet solution was discarded, rinsed with PBS for 3 times, and then photographed.

### 2.8 Statistical Methods

The computer program Statistical Package for the Social Sciences (SPSS) (V13, SPSS, 1 Chicago, IL) was used. Student t-tests were performed when comparing data of different groups.

## 3 Results

### 3.1 CPC particles inhibit the biological function of nucleus pulposus cells

**Figure 1:**
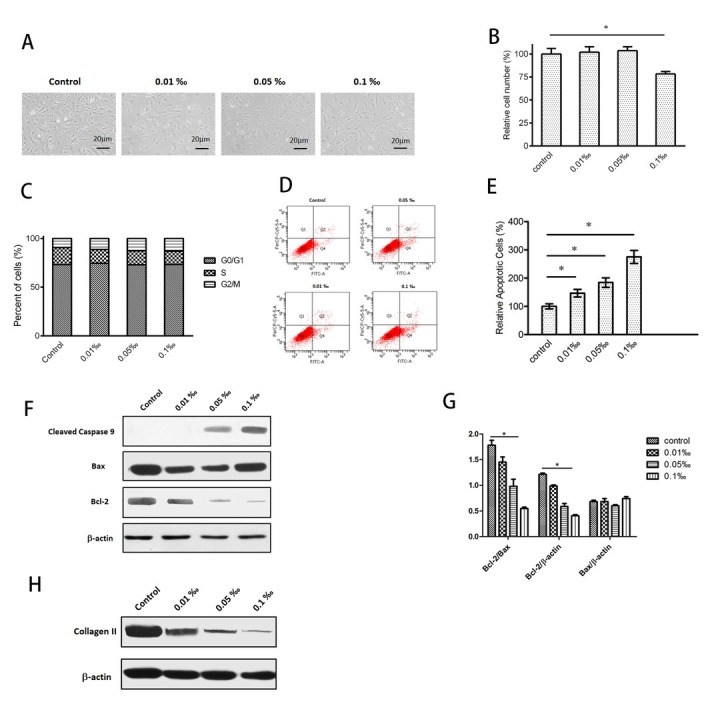
CPC particles inhibit the biological function of HNPCs. (**A**) Under the light microscope, HNPCs appeared to be shrunken after the treatment with CPC particles, especially when treated with a higher concentration. (**B**) Result of CCK-8 assay showed that the proliferation of cells was significantly inhibited at a higher CPC concentration (0.1‰ v/v, p<0.05). (**C**) Cell cycle distribution showed that NPCs were mostly arrested in G2 phase after CPC treatment. (**D, E**) Flow cytometry analysis indicated that the percent of apoptotic cells increased with CPC content (p<0.05). (**F**) Western blot indicated that the pro-apoptotic factors cleaved caspase 9 increased with CPC concentration. The Bax slightly increased whereas the anti-apoptotic factor Bcl-2 decreased**. (G)** Grey analysis showed that the Bcl-2/β-actin and Bcl-2/Bax values were significantly decreased in a dose dependent manner. The Bax/β-actin value showed a slight increase but there was no statistical significance. **(H)** Western blot showed that CPC treatment reduced the expression of type II collagen in a dose-dependent manner.

After 10 days of treatment, the morphological changes of the cells in each group were observed and compared under an optical microscope. It was found that the cells were shrunken and the particles increased after CPC treatment, and the cells in the group with higher concentration of CPC were obviously more shrunken with the cells became thinner and longer (Figure 1A). What’s more, the result of CCK-8 assay showed that the inhibitory effect of CPC particles on the proliferation of nucleus pulposus cells increased with the increase of particle concentration. CPC particles at low concentrations (0.01 and 0.05‰ v/v) had no significant effect on cell viability (p > 0.05), but a higher dose (0.1‰ v/v) significantly inhibited cell viability of HNPCs (p < 0.05) (Figure 1B). Cell cycle distribution analysis showed that with the increasing concentration of CPC, more and more cells were found to be stuck in G2 phase, indicating that the decrease of cell numbers in bone cement group was related to cell cycle arrest (Figure 1C). The result of flow cytometry analysis indicated that there were fewer apoptotic cells in the control group but more in the CPC group (Figure 1D, E). Additionally, Western blot analysis showed that, compared with the control group, CPC led to the expression of active Caspase 9 and Bax increase, and Bcl-2 decrease (Figure 1F). The grey analysis of the Western blot indicated that the Bax/ β-actin value showed a slight increase but there was no statistical significance, and the Bcl-2/β-actin and Bcl-2/Bax values were significantly decreased in a dose dependent manner (Figure 1G), which presented an apoptosis trend of HNPCs after being treated with CPC particles. After 10 days of treatment, the content of functional factors related to NPCs was detected by Western blot. It was found that Collagen II content in CPC group was significantly decreased, indicating that bone cement actually induced degeneration of HNPCs (Figure 1H).

### 3.2 CPC particles inhibit the ERK signaling pathway

**Figure 2:**
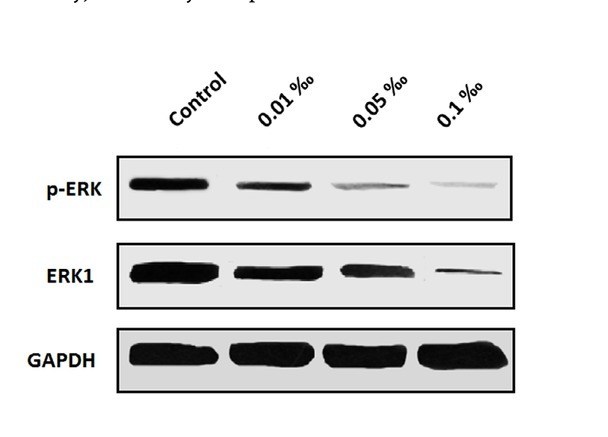
Western blot showed that both the total ERK protein level and the active form of ERK were significantly reduced after treated with CPC particles, indicating that CPC particles inhibited the ERK signaling pathway.

After 10 days of treatment, the phosphorylation level (active form) and total protein level of ERK protein in each group were detected by Western Blot. It was found that, compared with the control group, the ERK signaling pathway was significantly down-regulated in the bone cement group, in which the total ERK protein level and the active form of ERK were both significantly reduced (Figure 2). The ERK signaling pathway, also known as Ras/Raf/ MEK/ERK signaling cascades, consists of a 3 stage enzyme linked functional unit, namely Raf-MEK-ERK, which can be phosphorylated successively. ERK is the only downstream substrate of MEK; as such, the reduced content of ERK may show the inhibition of the ERK signaling pathway visually, caused by CPC particles.

### 3.3 Activation of ERK signaling pathway can reverse CPC-induced nucleus pulposus cells degeneration

**Figure 3:**
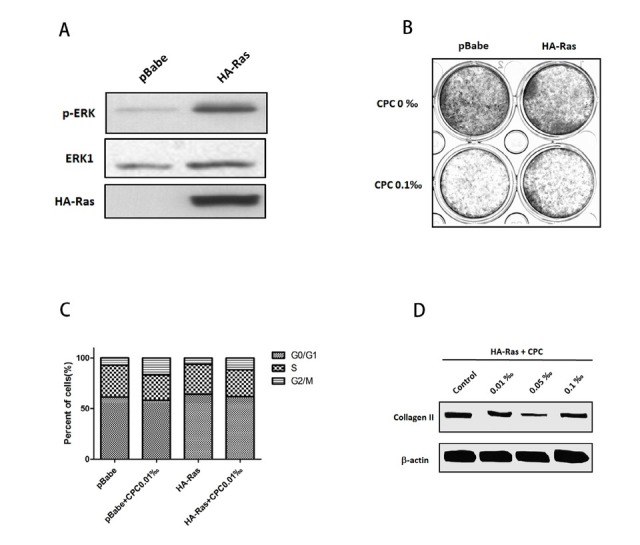
Activation of the ERK signaling pathway can reverse CPC-induced nucleus pulposus cells degeneration. (**A**) Western blot indicated that after Ras overexpression, the protein level of ERK-1 and p-ERK were significantly elevated, which meant that the ERK signaling pathway was activated. (**B**) Crystal violet staining showed that the inhibition of cell proliferation caused by CPC was partly reversed in HA-Ras group but still not as well as none-CPC groups. (**C**) Cell cycle distribution analysis showed that Ras overexpression reversed the G2 phase arrest caused by CPC. (**D**) Western blot indicated that after Ras overexpression, the content of type II collagen was not decreased so much as before because of the high dose of CPC (0.1‰ v/v) when ERK pathway had not been activated.

Through virus infection and puromycine screening, the stable Ras expression nucleus pulposus cell lines with HA markers were established. Western blot analysis showed that the constructed cells expressed Ras stably and could significantly activate the expression of ERK-1 and p-ERK proteins (Figure 3A). The results of the crystal violet experiment showed that the inhibition degree of CPC on cell growth was alleviated when the ERK pathway was activated (Figure 3B). Additionally, there was some recovery of G2-M cell cycle arrest induced by CPC after activation of ERK pathway, as was shown in the cell cycle distribution analysis (Figure 3C). What’s more, after the ERK signaling pathway was activated, the phenomenon of Collagen II down-regulation was greatly improved (Figure 3D). This indicated a significant improvement in the degeneration degree of HNPCs. All of the above showed that when the ERK signaling pathway was activated from upstream, the inhibition effects of CPC particles on HNPCs were weakened. As such, the CPC-induced HNPCs’ degeneration can be reversed by activating the ERK signaling pathway.

## 4 Discussion

### 4.1 The comparison between PMMA and CPC in IVD or HNPCs’ degeneration at the macroscopic and biological level

Indeed, CPC, as one of the most viable substitutes for the conventional PMMA, was very different from PMMA in several properties. Though the intradiscal cement leakage is less symptomatic or severe than other kinds of leakage, this doesn’t mean it does no harm to the vertebral disk in the long term. When we discuss their effects on IDD or the degeneration of HNPCs, we would like to divide the effects into the macroscopic level and the biological level.

From the macroscopic level, three of their properties in particular should be taken into consideration, which are the heat release during solidification, the damage of the endplate and the mechanical property. When it comes to the heat release, CPC has proven to produce much less heat than PMMA [4, 5]. There’s no doubt that the bone cement can reach a high cure temperature in a short time span after being injected into the vertebra, especially the PMMA. However, most studies indicated that if the bone cement is confined within the vertebra, the curing temperature will not be enough to cause obvious damage to surrounding tissues, even if PMMA is used [15]. Of course, we don’t deny that the heat will certainly be harmful to IVD if intradiscal leakage happens, especially during or shortly after the solidification of bone cement. Under that circumstance, the severity of the effect is not only related to the temperature, but the amount of leaked bone cement and the damage degree of endplates should also be taken into consideration. The leakage of bone cement to the disk often occurs through the end plate, which could result in damage to its normal structure. The vertebral endplate consists of hyaline cartilage weakly bonded to the perforated cortical bone of the vertebral body, and collagen fibers of the annulus and nucleus [22]. It’s an important structure for the metabolic transportation of NPCs [23]. So we suppose that when the leakage happens, the microcirculation of the disc is limited. As a result, anaerobic metabolism may happen and the accumulation of lactic acid and decreasing of PH may lead to disk degeneration [24]. From the available research data, we can’t recognize which kind of bone cement may do less harm to the endplate. When it comes to the mechanical property, studies have shown that the tensile and compression strength of normal human cancellous bone are 1-5 MPa and 4-12MPa, respectively, while those of PMMA are 49MPa and 114MPa [25]. The three-point bending strength of CPC is 10-15MPa and the strength of extension and compression is 10-15MPa, which are both far lower than PMMA [26]. Though there are studies indicating that the higher Young modulus of PMMA may cause a stress shielding effect, which may increase the incidence of adjacent vertebral fracture, there’s not enough evidence to show that the mechanical property change of the vertebra after being treated with bone cement might affect the IVD [25]. However, bone cement injected into the disc may increase the pressure in the disc space because the intervertebral disc is a closed structure without blood vessels. This in turn exerts abnormal stress on the nucleus pulposus cells and extracellular matrix, which may lead to disc degeneration [27, 28].

Regarding the biological level of whether or how bone cement particles may induce IDD, which is the main aim of our study, our group has previously done some research on the biological mechanism of how PMMA particles induce HNPCs’ degeneration [29]. That study revealed the role of PMMA particles in inducing IVD degeneration by downregulating CTGF through Hippo/YAP pathway. As the effect of CPC particles on HNPCs remains controversial or was observed to be not as obvious as PMMA [16], we carried out this study to explore whether and how CPC particles affect HNPCs biologically. Interestingly, our results proved that CPC particles could induce the HNPCs’ degeneration by blocking the activation of HNPCs’ ERK pathway. This could provide an explanation from the biological standpoint that CPC particles do have the capacity to induce IDD.

### 4.2 Our in vitro study indicated that CPC can affect HNPCs’ biological behavior and cell cycle, thus causing the intervertebral disc degeneration

The proliferation ability of cells was detected by cck-8 assay. The results showed that CPC particles with different concentrations had an effect on the activity of nucleus pulposus cells, and CPC particles with high concentrations had a more significant effect. The cell morphology also changed noticeably as the shape became thinner and longer, and there was an obvious wrinkling phenomenon. Our study also showed that CPC particles were involved in the regulation of the nucleus pulposus cell cycle. The result of flow cytometry indicated that cells were arrested in the G2 phase and the detection of apoptosis markers showed the apoptosis trend caused by CPC. Compared with the control group, cell adhesion molecules such as cadherin, selectin and integrin were down-regulated after CPC treatment, indicating that cells were prone to shed and die. Western results also showed that the expression of Caspase 9 protein increased significantly after CPC was added. CPC also increased the expression of Bax slightly (not significantly as was shown in the grey analysis), decreased the expression of Bcl-2, and reduced the ratio of Bcl-2 /Bax in HNPCs, thus turning on the “switch” to accelerate the apoptosis of NPCs. The detection of degeneration markers in our study confirmed that CPC did cause intervertebral disc degeneration. In our study, it was also found that Collagen II, the functional protein secreted by HNPCs, was significantly reduced after CPC treatment. Collagen II is concentrated in nucleus pulposus and gradually decreases outward. Collagen II has more hydroxylated proline, lysine and a large amount of hydroxylated galactose-glucose substitute, which is conducive to limiting the lateral growth of fibers and preventing collagenase digestion, so as to stabilize the disc [30, 31].

### 4.3 The role of ERK signaling pathway in intervertebral disc degeneration

There have already been many studies describing the relationship between the ERK signaling pathway and NPCs degeneration. Zheng et al. found leptin could induce the proliferation of human NPCs via MEK/ERK pathways [32]. It’s still controversial whether the activation of the ERK signaling pathway can induce or inhibit nucleus pulposus cell degeneration. We suppose that the ERK pathway is one kind of ‘stress-react’ pathway that can be activated when NPCs face the risk of degeneration. Makarand et al. confirmed that NPCs upregulated the MEK/ERK and PI3K/Akt signaling pathway under hypoxic conditions and resisted apoptosis [19]. Shuai L et al. found that Autophagy attenuates compression-induced apoptosis of human nucleus pulposus cells via MEK/ERK/ NRF1/Atg7 signaling pathways during intervertebral disc degeneration [33]. What’s more, autophagy and apoptosis have been confirmed by scientists in that they can respond to similar stress stimuli and can both be involved in intervertebral disc degeneration [34, 35, 36, 37, 38]. Other in vivo studies showed that the ERK signaling pathway can be activated under hypertonic stress and cause apoptosis of rabbit NPCs. The latest study indicated that the expression of ERK1/2 was decreased with overexpression of miR-155 in normal nucleus pulposus cells [39]. All these factors seems to prove that the ERK signaling pathway is more like a ‘stress-react’ pathway that can be activated to inhibit the NPCs’ degeneration.

In conclusion, our study found that CPC particles have a negative effect on HNPCs, which was mainly reflected in cell growth and the cell cycle. After activation of the ERK signaling pathway, the effect of CPC on cell growth and the cell cycle was significantly reduced as well as the content of Collagen II, which could reflect that the degree of HNPCs’ degeneration was up-regulated significantly. We can draw the conclusion that CPC can probably block the activation of HNPCs’ ERK pathway, thus causing the HNPCs’ degeneration. Our study gave an explanation from the biological standpoint that CPC particles do have the capacity to induce IDD.
